# Differences in antimicrobial consumption, prescribing and isolation rate of multidrug resistant *Klebsiella pneumoniae*, *Pseudomonas aeruginosa* and *Acinetobacter baumannii* on surgical and medical wards

**DOI:** 10.1371/journal.pone.0175689

**Published:** 2017-05-03

**Authors:** Vladimir Zivanovic, Ljiljana Gojkovic-Bukarica, Radisav Scepanovic, Teodora Vitorovic, Radmila Novakovic, Nenad Milanov, Zoran Bukumiric, Biljana Carevic, Jasmina Trajkovic, Jovana Rajkovic, Vladimir Djokic

**Affiliations:** 1 University Hospital Center “Dr. Dragisa Misovic-Dedinje”, Belgrade, Serbia; 2 Institute of Pharmacology, Clinical Pharmacology and Toxicology, Medical faculty, University of Belgrade, Belgrade, Serbia; 3 Department of Medical Statistics, School of Medicine, University of Belgrade, Belgrade, Serbia; 4 Department of Hospital Epidemiology, Clinical Center of Serbia, Belgrade, Serbia; Azienda Ospedaliera Universitaria di Perugia, ITALY

## Abstract

In order to provide guidance data for clinically rational use of an antibiotics consuption, prescribing and prevalence of multidrug resistant (MDR) *Klebsiella pneumoniae*, *Pseudomonas aeruginosa* and *Acinetobacter baumannii* were monitored on the surgical (S) and medical (M) wards of the University Hospital Center “Dr. Dragisa Misovic-Dedinje” (Belgrade, Serbia), in the study period from 2012 to 2015. Appropriateness of antimicrobial use was evaluated using the Global-Prevalence Survey method designed by the University of Antwerp. The percentages of MDR pathogens relative to the total number of isolates of *K*. *pneumoniae* and *P*. *aeruginosa* were higher on the S (86.2% and 49.1%) than on the M (63.2% and 36.9%) wards. The percentage of MDR *A*. *baumannii* was not different between S (93.7%) and M (79.5%) wards. An overall antibiotics consumption (defined daily doses/100 bed-days) during study was 369.7 and 261.5 on the S and M wards, respectively. A total of 225 prescriptions of antimicrobials were evaluated in138 adults admitted to wards on the day of the survey. The percentage of antimicrobials prescribed for prophylaxis on the M and S wards were 0% and 25%, respectively. Therapies were more frequently empiric (S, 86.8% and M, 80%). The percentages of medical errors on the S and M wards were 74.6% and 27.3%, respectively. The quality indicators for antibiotic prescribing on the S and M wards were as follows: the incorrect choice of antimicrobials (35.6% vs. 20.0%), inappropriate dose interval (70.6% vs. 16.9%) or duration of therapy (72.5% vs. 23.1%), a non-documented stop/review data (73.6% vs. 16.9%) and divergence from guidelines (71.9% vs. 23.1%). Treatment based on biomarkers was more common on the M wards as compared to the S wards. The increasing prevalence of MDR pathogens, a very high consumption and incorrect prescribing of antimicrobials need special attention, particularly on the S wards.

## Introduction

The misuse and overuse of antibiotics are widespread, not only in poor and developing countries, but also worldwide. The inappropriate use of antibiotics has led to the rise in antimicrobial resistance [1]. Infections caused by multi-drug resistant microorganisms (MDR), often do not respond to conventional therapy and result in a longer duration of illness and higher risk of death. Hospital-acquired infections, especially "big four" (surgical site infections, pneumonia, blood stream and urinary tract infections) are commonly caused by extended-spectrum beta-lactamase (ESBL) producing Enterobacteriaceae (*Escherichia coli*, *Klebsiella pneumoniae and Proteus mirabilis*) and non-fermenting gram-negative *Pseudomonas aeruginosa* and *Acinetobacter baumannii* [2].

According to the annual Report of the Central Asian and Eastern European Surveillance of Antimicrobial Resistance (CAESAR) network for 2013, among 30 EU-countries and some countries from ex-Yugoslavia, Serbia had the highest prevalence of *K*. *pneumoniae* resistant to the third-generation cephalosporines (88.5%), and a very high prevalence of *K*. *pneumoniae*, *P*. *aeruginosa* and *A*. *baumannii* resistant to carbapenems (31.4%, 44.6% and 92.1%, respectively) [3]. A total antibiotic consumption in 2011 (expressed in number of DDD per 1000 inhabitants per day) was higher in Serbia (26) than collectively in Croatia and Bosnia and Herzegovina (around 20), but still lower than collectively in Greece and in Montenegro (around 40) [4]. As antibiotic consumption in EU- and non-EU countries differs significantly from one country to another, it is important to provide national, region and country-specific quality targets to improve antibiotic use. Despite its importance, there is a very little available information about antimicrobial consumption and the prevalence of resistance in hospitals of Serbia.

In order to provide guidance data for clinically rational use of antibiotics, the distribution, resistance of common clinical isolates and consumption in the adults were analyzed on the surgical (S) and medical (M) wards of the University Hospital Center “Dr. Dragisa Misovic-Dedinje” (Belgrade, Serbia) from 2012 to 2015. The Global Point Prevalence Surveys (G-PPS) method developed by the Laboratory of Medical microbiology of the University of Antwerp, Belgium was used for monitoring prescribing [5]. This tool provided quantifiable outcomes measures to assess and compare quantity and quality of antibiotic prescribing in hospitalized patients in different wards.

## Materials and methods

The study presented here is the sum of two different studies: i) trends in antimicrobial consumption and in the proportion of MDR pathogens conducted from 2012 to 2015 on the S and M wards of the University Hospital Center “Dr. Dragisa Misovic-Dedinje” (Belgrade, Serbia) and ii) point-prevalence survey of antimicrobial consumption and appropriateness of prescribing performed in a single day.

### Hospital setting

Antibiotics consumption and antimicrobial resistance were monitored on the S and M wards in the study period from 2012 to 2015. The results were presented for every year. Bed capacity of the whole University Hospital Center “Dr. Dragisa Misovic-Dedinje” was 387, with 46 and 91 on the S and M wards respectively. The S wards consist of departments for laparoscopic abdominal surgery, laparoscopic urological surgery, urology and an intensive care unit (ICU). The M wards consist of departments for geriatric, pulmonology, hematology, endocrinology, noninvasive cardiology, gastroenterology and ICU. Other wards of the University Hospital Center were not included in this analysis (e.g. psychiatry, neurology, pediatric and gynecology and obstetrics). Each ward within the University Hospital Center “Dr. Dragisa Misovic-Dedinje” is located in a separate building.

At the beginning of 2012, different interventions to restrict and improve antibiotic use were introduced in our institution: monitoring and evaluation of MDR on each ward and ICU on monthly basis; the local guide for prophylactic and therapeutic use of antibiotics in hospital, based on the local map of resistance and consistent with national and international guides [6–10]; evaluation of the antibiotics consumption on the basis of antibiotic purchasing data from the hospital information system; the control of prescription of some antibiotics, and the organization of personnel training on the use of antibiotics in hospital settings.

Since 2010, the infection-control measures consistent with the Center for Diseases Control and Prevention and the Hospital Infection Control Practice Advisory Committee guideline and WHO Guidelines on Hand Hygiene in Health Care have been adopted in our University Hospital Center [11, 12]. Standard precautions, applied to all patients, include hand hygiene according to pre-specified guidelines, the use of personal protective equipment, respiratory hygiene, safe injection practice (safety needle—BD Eclipse™), the use of mask for catheter insertion and lumbar puncture procedures, safe handling of contaminated equipment (including plasma H_2_O_2_ treatment), textiles and laundry as well as routine cleaning, disinfection of environmental surfaces (space disinfection by means of H_2_O_2_) and safe waste removal. Other measures are as follows: cohorting, screening of all patients before admission to the ICUs, decolonization, personnel and environmental screening and personal training.

### Antimicrobial testing

Routine screening of microbiological resistance/sensitivity is carried out by bacteriological laboratory of the University Hospital Centre "Dr. Dragisa Misovic-Dedinje" for all cases requiring antibiotic therapy, except for prophylactic use. Bacterial strains were isolated from the clinical material (fluids from the intra-abdominal infections, incisions, abscesses, fistulas, blood, urine, sputum, etc.) obtained from the hospitalized patients, and susceptibility to antibiotics was assessed by diffusion and standard microdilution methods. Identification and susceptibility testing was performed using the Vitek 2 system (bioMerieux, Marcy I'Etoile, France) in microbiological laboratory of our hospital. The epidemiological cut-off values for reduced susceptibility were defined according to Clinical and Laboratory Standards Institute (CLSI, MS100-S21) [13]. If cut-off values were not available, the European Committee on Antimicrobial Susceptibility Testing (EUCAST) recommendations were used [14]. We used U.S. Food and Drug Administration (FDA)-approved breakpoints for tigecycline to determine *A*. *baumannii* and *P*. *aeruginosa* susceptibility to this drug [15]. Bacterial resistance was reported as the percentage of total isolates showing multidrug resistance (MDR, the isolate is non-susceptible to at least 1 agent in ≥ 3 antimicrobial categories) [16]. The classes used to define MDR isolates of *K*. *pneumoniae* were aminoglycosides (amikacin), beta-lactams (ampicillin, amoxicillin-clavulanic acid, cefepime, ceftriaxone, or piperacillin-tazobactam), carbapenems (imipenem/meropenem), fluoroquinolones (levofloxacin) and glycylcyclines (tigecycline). The classes used to define MDR *P*. *aeruginosa* isolates were aminoglycosides (amikacin), beta-lactams (cefepime, ceftazidime, or piperacillin-tazobactam), carbapenems (imipenem/meropenem), and fluoroquinolones (levofloxacin). The classes used to define MDR *A*. *baumannii* isolates were aminoglycosides (amikacin), beta-lactams (cefepime, ceftazidime, ceftriaxone, or piperacillin-tazobactam), carbapenems (imipenem/meropenem) and fluoroquinolones (levofloxacin). The burden of resistance for each antibiotic was calculated as the percentage of all "Resistant" + "Intermediate Resistant" results among all tested isolates from all patients samples [12]. Repeated isolates were excluded from data if the same organism was grown in the same patient in 15-day interval. The surveillance cultures were not included in the analyses. WHONET 5.6 software was used for analysis of susceptibility rates in different year and wards.

In patients with history of previous hospitalization and/or treatment by antimicrobial agents who developed diarrhea, the diagnosis of *C*. *difficile* infection was established using the VIDAS^®^ *C*. *difficile* Toxin A & B (CDAB) test, based on the ELFA (Enzyme-Linked Fluorescent Assay) technique (bioMerieux).

### Antimicrobial consumption

Four years of antimicrobial consumption were evaluated on the basis of the antibiotic purchasing data provided from the hospital information system. The amount of antimicrobial drug in grams was converted in number of defined daily doses (DDD)/100 bed-days (DBD) using the WHO classification system ATC. Following the implementation of restriction policy, the prescription of some antibiotics (piperacillin/tazobactam, ampicillin/sulbactam, carbapenems, amikacin, linezolid, tigecycline) could be controlled. These antibiotics could only be prescribed after the approval granted by the local Committee for controlof antimicrobial prescription consisting of the specialists in hospital infection, clinical pharmacologists and clinical microbiologists. However, ampicillin/sulbactam, cefepime, ticarcillin-clavulanic acid, tobramycin, fosfomycin, doripenem and colistin were excluded from our statistical analysis because they were not available on the Serbian market in 2012 and/or in 2013.

### Antimicrobial prescribing

Point prevalence study was performed voluntarily in our tertiary care hospital using the Global Prevalence Survey (G-PPS) method. A web-based application was used for data-entry, validation and reporting as designed by the University of Antwerp [5]. Data were collected for all patients admitted to the M and S wards and received some antimicrobial on the day of the survey (from 8 a.m. 1^st^ June, 2015 to 8 a.m. 2^nd^ June, 2015). Relevant variables included age, gender, department of hospitalization, duration of hospitalization, reasons and indications for treatment and microbiological data. Antimicrobial agents were analyzed for dose, route of administration and duration of therapy/prophylaxis. The analyzed Quality Indicators (QIs) for antibiotics use were as follows: reason in notes, compliance with guidelines, documented stop/review data and target prescribing of antibiotics. In addition, we analyzed antimicrobial prescription based on biomarkers (C-reactive protein rapid test, CRP, procalcitonin, PCT and the number of leukocytes). The appropriateness of each prescription was evaluated by specialists in hospital infection, clinical microbiologists and clinical pharmacologists. A local guideline of antibiotics prescription was adapted to local microbiological map and in compliance with the international guidelines, to judge the appropriateness of the antimicrobial therapy and define medical errors [6–10]. The criteria used to judge the adequateness of the antimicrobial therapy/prophylaxis were described previously by Cusini et al., and they are as follows: appropriate decisions if all criteria of correct antimicrobial use are fulfilled, inappropriate indications, inappropriate choice, inappropriate application (inappropriate dosage, timing, route of administration and duration of therapy/prophylaxis), divergence from guidelines and insufficient data to judge the appropriateness of antimicrobial use [17].

### Ethics

The study has been approved by the local Ethics Committee of the University Hospital Center “Dr. Dragisa Misovic-Dedinje”, (Belgrade, Serbia). The Ethics Committee has decided that patient’s formal consent will not be required because this study was a quality control project.

### Statistics

The results were presented as frequency (percent), median (range) and mean ± SD. For parametric data independent samples t-test was used to test differences between the groups. Mann-Whitney U test was used for numeric data with non-normal distribution and ordinal data. Chi-square test or Fisher's exact test was used to test differences between the nominal data (frequencies). All p values less than 0.05 were considered significant. Statistical data analysis was performed using the IBM SPSS Statistics 22 (IBM Corporation, Armonk, NY, USA).

## Results

### Patients characteristics, indications for antimicrobial therapy and bacterial isolates

Annual distribution of isolates, patients numbers and median duration of hospital stays are shown in Table 1. A total number of isolates on the S and M wards was 1953 and 5984, respectively. Strains were separated within four years (2012–2015). During the whole interval of study the median duration of hospital stays on the M (10.0, 9.5, 9.2 and 9.3 days) was significantly longer (p < 0.05) than on the S (3.0, 2.8, 2.8 and 3.1 days) wards. A total number of patients on the S and M wards were 11,686 and 17,105, respectively. The mean age on the S wards was 55.7 years (range: 18–85) and 8005 (68.5%) were males. The mean age on the M wards was 70.3 years (range: 18–92) and 8125 (47.5%) were males.

**Table 1:** Annual distribution of isolation rates and patients on surgical and medical wards.

Ward	Variables	2012	2013	2014	2015
**Surgical wards**	No. of total isolates	485	436	524	508
	No. of patients	4117	4431	4206	4351
	Mean days of hospital stay*	3.0	2.8	2.8	3.1
**Medical wards**	No. of total isolates	1597	1468	1465	1454
	No. of patients	2700	3038	2930	3018
	Mean days of hospital stay*	10.0	9.5	9.2	9.3

* During the whole interval of study the median duration of hospital stays on the M was significantly longer than on the S wards (p < 0.05).

The main indications for antimicrobial therapy during the whole interval of study are summarized in Table 2. The urinary tract infections (n = 2671, 74.3%), gastrointestinal tract infections (n = 725, 20.1%) and skin and soft tissue infections (n = 95, 2.6%) were the most frequent diagnoses on the S ward. The urinary tract infections (n = 2192, 34.9%), respiratory tract infections (n = 2002, 31.9%) and sepsis/bacteremia (n = 836, 13.3%) were the most frequent diagnoses on the M ward. The indications for antimicrobials therapy varied widely between the different wards (p < 0.001). In 208 patients, with a history of previous hospitalization and/or treatment with the antimicrobial agents who developed diarrhea, the diagnosis of *C*. *difficile* infection was established in 48 and 160 patients from S and M ward, respectively. The percentage of patients with *C*. *difficile* infection relative to the total number of indications for antimicrobial therapy was higher on the M (2.5% vs. 1.3%) than on the S wards (p < 0.001).

**Table 2:** Main indications for antimicrobial therapy from 2012 to 2015.

Indications	Surgical wards No. (%)	Medical wards No. (%)
**Respiratory tract infection**	35 (1)	2002 (31.9)
**Sepsis, bacteremia**	23 (0.6)	836 (13.3)
**Gastrointestinal tract infection**	725 (20.1)	681 (10.8)
**Urinary tract infection**	2671 (74.3)	2192 (34.9)
**Skin and soft tissue infection**	95 (2.6)	83 (1.3)
**Fever of unknown origin**	0 (0)	330 (5.3)
***C*. *difficile* infection**	48 (1.3)	160 (2.5)
**Total**	**3597 (100)**	**6284 (100)**

*E*. *coli* (15.1% and 26.2%), *Enterococcus spp*. (16.5% and 14.5%) and *K*. *pneumoniae* (14.1% and 8.9%) were the most common isolates on the S and M wards (Table 3). The percentages of clinical isolates relative to the total number of isolates differed among the wards (p < 0.001).

**Table 3:** Types and frequency of appearance of bacterial strains from 2012 to 2015.

	No. (%)	No. (%)
Isolates	Surgical wards	Medical wards
*Escherichia coli*	295 (15.1)	1568 (26.2)
*Klebsiella pneumoniae*	276 (14.1)	532 (8.9)
*Proteus mirabilis*	150 (7.7)	467 (7.8)
*Pseudomonas aeruginosa*	228 (11.6)	317 (5.3)
*Acinetobacter baumannii*	32 (1.6)	297 (5)
*Enterobacter spp*.	35 (1.8)	197 (3.3)
*Staphylococcus aureus*	57 (2.9)	494 (8.3)
*MRSA*	13 (0.7)	116 (1.9)
*Staphylococcus epidermidis*	221 (11.3)	209 (3.5)
*Enterococcus spp*.	317 (16.5)	841 (14.1)
*VRE*	23 (1.2)	72 (1.2)
*Other*	306 (15.7)	874 (14.6)
**All**	**1953 (100)**	**5984 (100)**

### Annual distribution, resistance of *K*. *pneumoniae*, *P*. *aeruginosa* and *A*. *baumannii* strains and trends in antibiotic consumption

**Fig 1:**
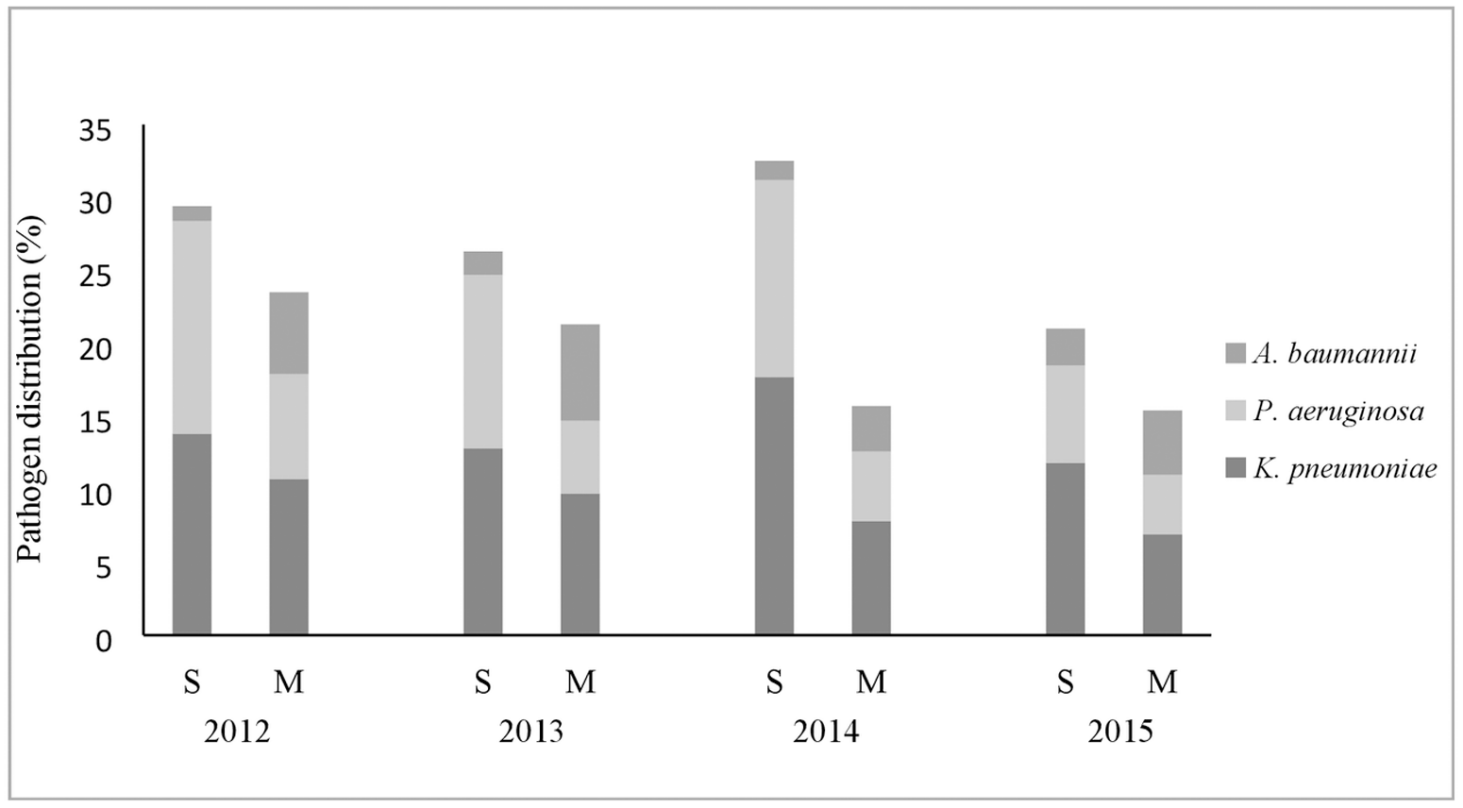
Annual distribution of *Klebsiella pneumoniae*, *Pseudomonas aeruginosa* and *Acinetobacter baumannii* strains isolated on surgical (S) and medical (M) wards from 2012 to 2015.

Annual frequency expressed as percentages of isolated *K*. *pneumoniae*, *P*. *aeruginosa* and *A*. *baumannii* strains were not significantly different during study period on both wards (Fig 1). A total number of *K*. *pneumoniae*, *P*. *aeruginosa* and *A*. *baumannii* strains isolated on the S wards was 276/1953 (14.1%), 228/1953 (11.6%) and 32/1953 (1.6%), respectively. A total number of *K*. *pneumoniae*, *P*. *aeruginosa and A*. *baumannii* strains isolated on the M wards was 532/5984 (8.9%), 317/5984 (5.3%) and 297/5984 (4.9%), respectively. The percentages of isolates of *K*. *pneumoniae* and *P*. *aeruginosa* relative to the total number of isolates were lower on the M than on the S wards (p < 0.001, both), but percentage of isolates of *A*. *baumannii* was higher on the M than on the S wards (p < 0.001).

Resistance rate of selected pathogens was shown in Table 4. *K*. *pneumoniae*, *P*. *aeruginosa* and *A*. *baumannii* strains from S showed higher resistance rate to carbapenems (imipenem) than the isolates from the M wards (p < 0.001, p < 0.001 and p < 0.05, respectively), but they displayed comparable resistance rate to cefepime (p > 0.05, all). The resistance rate of *K*. *pneumoniae* strains to ceftazidime was higher on the S than on the M wards (p < 0.001).

**Table 4:** Resistance rate (%) of *Klebsiella pneumoniae*, *Pseudomonas aeruginosa* and *Acinetobacter baumannii* strains isolated from 2012–2015.

Species Total no.	Antimicrobial agent	Surgical ward (%)	Medical ward (%)	p
***Klebsiella pneumoniae*** **S wards, 36** **M wards, 532**	Ceftazidime Cefepime Imipenem	85.8% 50.9% 42.2%	61.9% 44% 8.6%	<0.001 0.078 <0.001
***Pseudomonas aeruginosa*** **S wards, 228** **M wards, 317**	Ceftazidime Cefepime Imipenem	19.5% 19.7% 51.1%	21.5% 19.4% 25.1%	0.539 0.886 <0.001
***Acinetobacter baumannii*** **S wards, 32** **M wards, 97**	Ceftazidime Cefepime Imipenem	93.8% 93.8% 93.8%	85.7% 80.5% 77.1%	0.354 0.099 0.039

**Fig 2:**
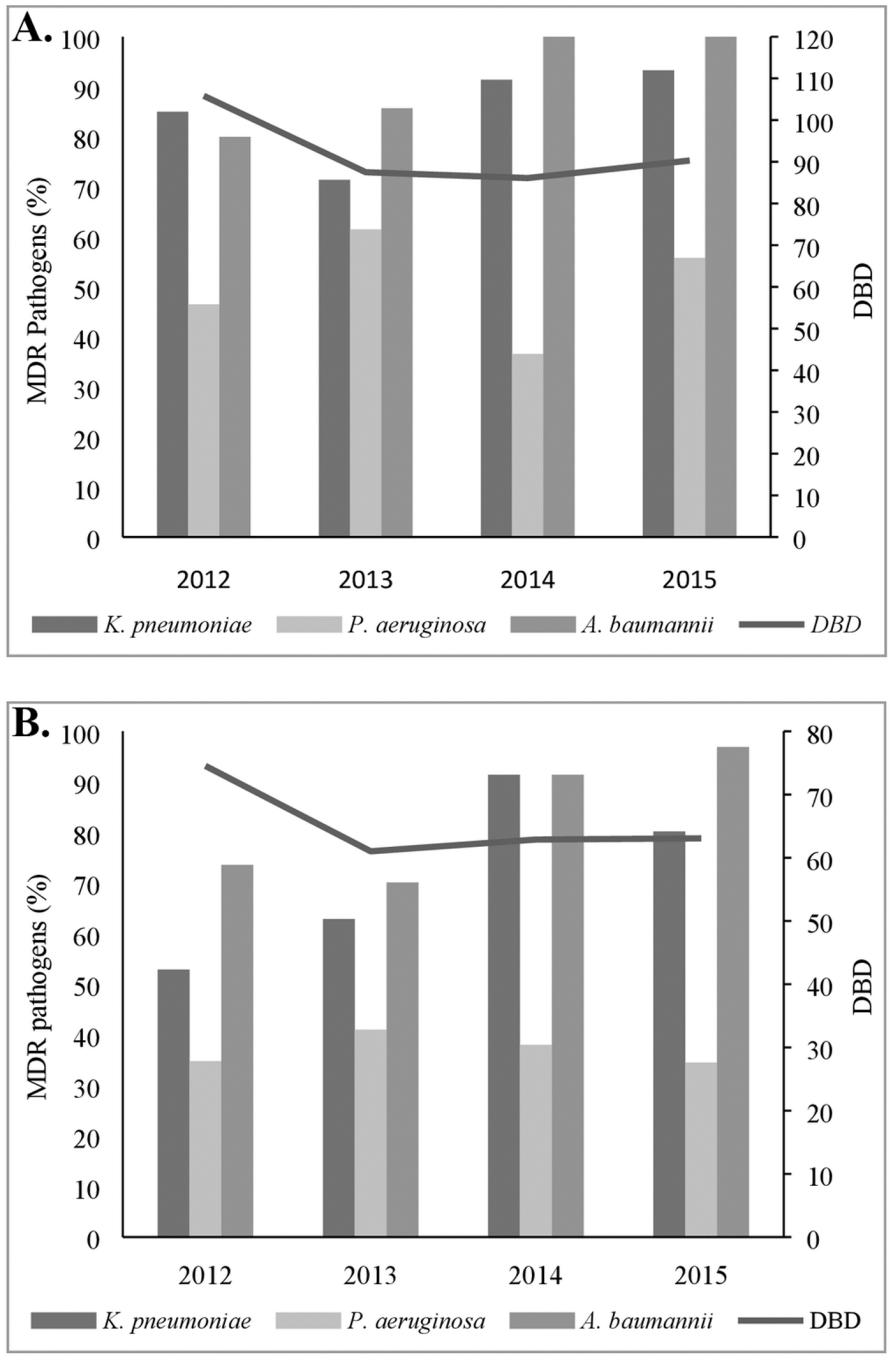
Association between antimicrobial consumption and isolation rate of multi drugs resistant *K*. *pneumoniae*, *P*. *aeruginosa* and *A*. *baumannii* at surgical (A) and medical (B) wards.

The percentages of MDR *K*. *pneumoniae*, *MDR P*. *aeruginosa* and MDR *A*. *baumannii* on the S ward were 86.2% (238/276), 49.1% (112/228) and 93.7% (30/32), respectively (Fig 2A). The percentages of MDR *K*. *pneumoniae*, *MDR P*. *aeruginosa* and MDR *A*. *baumannii* on the M ward were 63.1% (336/532), 36.9% (117/317) and 79.5% (236/297), respectively (Fig 2B). The percentages of MDR *K*. *pneumoniae* and MDR *P*. *aeruginosa* were significantly lower on the M than on the S wards (p < 0.001, p = 0.006). The percentage of isolates of MDR *A*. *baumannii* was higher on the S than on the M wards, but not statistically significant (p = 0.086). There was no difference in the annual frequency of MDR pathogens on both wards.

Trends in antibiotic consumption according to all tested antibiotics recommended for therapy and prophylaxis on S and M wards are shown in Fig 2A and 2B. In 2012, 2013, 2014 and 2015, total antibiotics consumption expressed as DBD was significantly higher (p = 0.003) on S (105.8, 87.5, 86.1 and 90.3, respectively, Fig 2A) than on the M wards (74.5, 61.9, 62.9 and 63.1, respectively, Fig 2B). The highest consumption of all antibiotics, on both hospital wards, was in 2012 (but not statistically significant).

**Fig 3:**
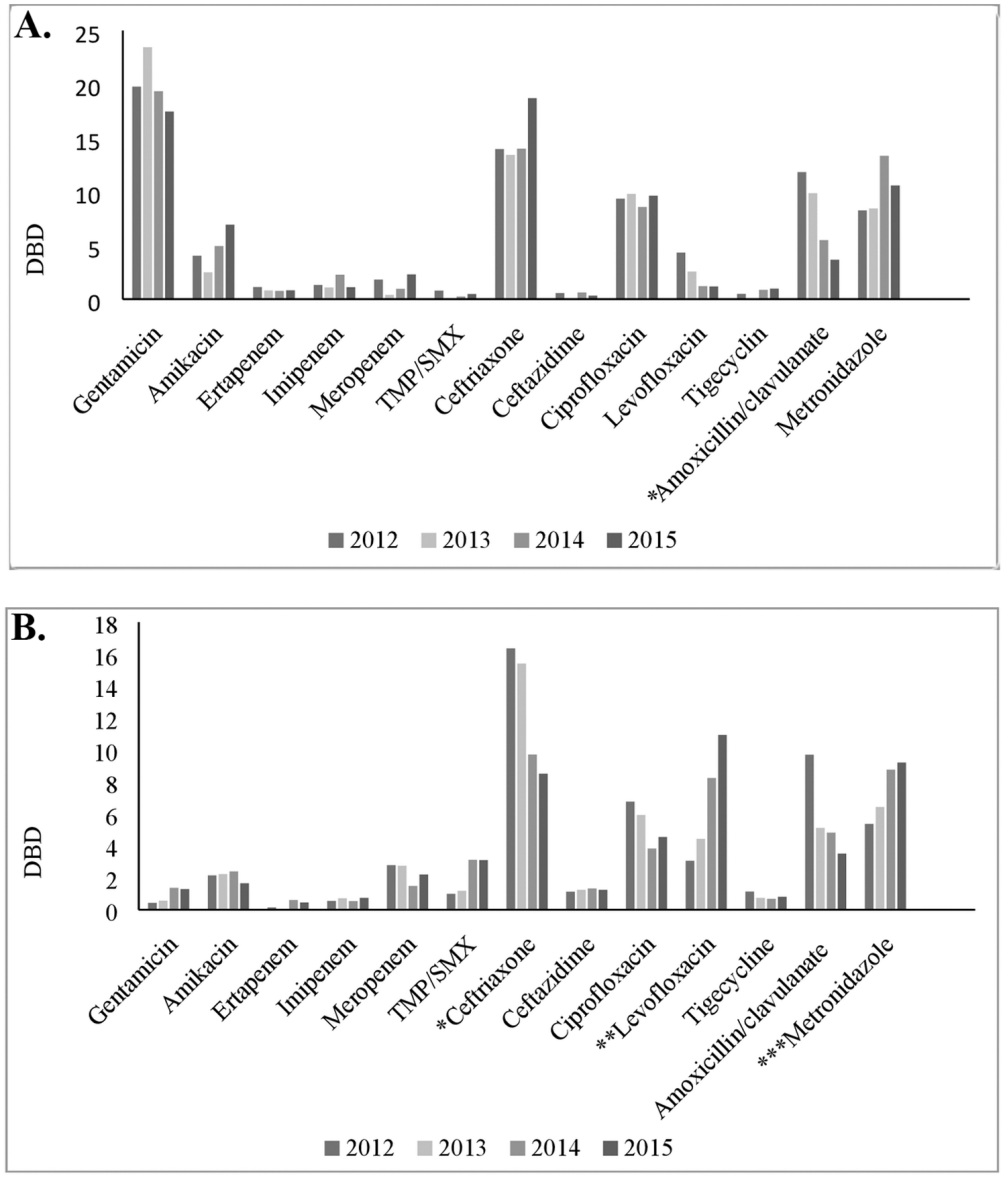
Trends of antibiotic consumption by individual drugs on surgical (A) and medical (B) wards from 2012 to 2015. A. *There was a statistically significant decrease in the use of amoxicillin/clavulanate (b = -2.881; p = 0.014). B. *There was a statistically significant decrease in the use of ceftriaxone (b = -2.925; p = 0.047). **There was a statistically significant increase in the use of levofloxacin (b = 2.745; p = 0.014). ***There was a statistically significant increase in the use of metronidazole (b = 1.385; p = 0.029).

According to total DBD, five most frequently used antimicrobials on the S wards from 2012 to 2015 were gentamicin (19.8, 23.5, 19.4 and 17.5, respectively), ceftriaxone (13.9, 13.4, 14.1 and 18.7, respectively), metronidazole (8.3, 8.4, 13.4 and 10.6, respectively), ciprofloxacin (9.4, 9.8, 8.6 and 9.6), and co-amoxiclav (11.8, 9.9, 5.5 and 3.7, respectively) (Fig 3A). The significant (b = -2.881, p = 0.014) decrease of consumption of co-amoxiclav was observed during the whole study time interval. According to total DBD, top five most common antimicrobials prescribed from 2012 to 2015 on the M wards were ceftriaxone (16.4, 15.4, 9.7 and 8.5, respectively), metronidazole (5.4, 6.4, 8.8 and 9.2, respectively), levofloxacin (3.1, 4.4, 8.2 and 10.9, respectively), co-amoxiclav (9.7, 5.1, 4.8 and 3.5, respectively) and ciprofloxacin (6.8, 5.9, 3.8 and 4.6, respectively). During the whole study time interval, the significant decrease of ceftriaxone was observed (b = -2.925; p = 0.047), whereas the use of levofloxacin and metronidazole was increased (b = 1.385; p = 0.029). In addition, the use of co-amoxiclav was decreased, but not statistically significant (Fig 3B). No correlation was observed between the antimicrobial use and the isolation rate of MDR pathogens.

### Prescribing tested by G-PPS methodology

The prescribing of antimicrobial was tested by G-PPS. We evaluated 225 (S, n = 160 and M, n = 65) prescriptions of the antimicrobial therapy in 138 adults admitted to hospital on the S (n = 92) and M (n = 46) wards on the day of the survey. The mean age (S, 65.1±14.4 and M, 77.0±12.8 years) and the mean duration of hospitalization (S, 5 days and M, 15 days) on the S and M wards were significantly different (p < 0.001 both). As expected, parenteral administration of antimicrobials was higher on the S (82.5%) than M (56.9%) wards (p < 0.001). Therapies were more frequently empiric on both wards (S, 86.8% and M, 80%, p = 0.198).

Table 5 lists the reasons for antibiotics prescribing. Percentage of antimicrobials prescribed for medical or surgical prophylaxis on the M and S wards was 0% and 25%, respectively. The percent of a single dose prophylaxis, one day and more than one day prophylaxis was 2.5%, 13.8% and 58.8%, respectively. The indications for antimicrobial therapy varied between different wards because of differences in underlying diseases of patients hospitalized on these wards (Table 5). The percent of antimicrobials prescribed for community- acquired infections and hospital acquired infections was significantly higher (p < 0.001) on the M wards. Overall, site infections and intervention -related infections (catheter- related blood stream infection, catheter- related urinary tract infection and ventilator associated pneumonia) were the most frequent diagnoses on the S wards.

**Table 5:** Reasons for antibiotics prescribing obtained by Global-Point Prevalence Survey method.

Reasons for antibiotics prescribing	Surgical wards No. (%) of prescriptions	Medical wards No. (%) of prescriptions	p
**Prophylaxis single dose**	4 (2.5)	0	0.327
**Prophylaxis one day**	22 (13.8)	0	<0.001
**Prophylaxis > one day**	94 (58.8)	0	<0.001
**CAP**^1^	20 (12.5)	46 (70.8)	<0.001
**HAP**^2^			
**Surgical site infections**^3^	7 (4.4)	0	0.197
**Intervention related infections**^4^	4 (2.5)	0	0.327
***C*.*difficile*-associated diarrhea (CDAD)**^5^	3 (1.9)	2 (3.1)	0.628
**HAP**^6^	6 (3.8)	13 (20)	<0.001
**Unknown**	0	4 (6.2)	0.007
**Total**	**160 (100)**	**65 (100)**	

^1^Community acquired pneumonia.

^2^Hospital acquired infections.

^3^Infections within 30 days after the operation or 1 year after the implant surgery.

^4^Catheter -related blood stream infection, catheter-related urinary tract infection and ventilator associated pneumonia.

^5^>48 h post admission or <30 days after discharge from previous admission episode.

^6^Hospital acquired pneumonia.

Table 6 shows an overview of the evaluation of antimicrobial use appropriateness on different wards. A total number of medical errors was highly significantly lower on the M wards compared to S wards (27.3% and 74.6%, p < 0.001). The divergence from guidelines was much higher concern on the S (72.9%) in relation to M wards (23.1%). Nevertheless, the non-documented indication, lack of reasons in notes, wrong dose and wrong route of administration were more often recorded on the M wards. Treatment based on biomarkers (CRP, PCT and the count of leucocytes) was more common on the S wards (p < 0.001, p < 0.001 and p = 0.021, respectively).

**Table 6:** Quality of antimicrobial prescribing obtained by Global-Point Prevalence Survey method.

Quality indicators	Surgical ward No. (%)	Medical ward No. (%)	p
**Indication documented** **Reason in notes**	156 (97.5) 156 (97.5)	55 (84.6) 55 (85.9)	0.001 0.001
**Guideline compliant**	45 (28.1)	50 (76.9)	<0.001
**The reasons for non-compliance** **- Wrong choice** **- Wrong dose** **- Wrong dose interval**	57 (35.6) 3 (1.9) 113 (70.6)	13 (20.0) 10 (15.4) 11 (16.9)	0.022 <0.001 <0.001
**Treatment based on biomarkers**			
**CRP**^a^ **PCT**^b^ **-Leucocytes**	64 (40) 39 (24.4) 148 (92.5)	63(96.9) 32(49.2) 65(100)	<0.001 <0.001 0.021
**Total no. of prescriptions**	**160 (100)**	**65 (100)**	

^a^C-reactive protein.

^b^Procalcitonin

During G-PPS, gentamicin (68/160, 42.5%), ceftriaxone (28/160, 17.5%), ciprofloxacin (22/169,13.8%), amikacin (14/160, 8.8%) and co-amoxiclav (9/160, 5.6%), were five most common prescribed antimicrobials on the S wards, while five most common antimicrobials prescribed on the M wards were: ciprofloxacin (17/65,26.2%), ceftriaxone (12/65,18.5%), levofloxacin (10/65, 15.4%), metronidazole (9/65, 13.8%) and azithromycin (4/65, 6.2%).

## Discussion

The last Surveillance Reports of the European Center for Disease Prevention and Control found that antibiotic resistance rates of MDR *K*. *pneumoniae* in 2014 varied markedly between the EU-countries (Iceland, 0%, Slovenia, 18.9%, Croatia, 30.9%, Bulgaria, 41.7, Romania, 56.0%, Greece, 56.8%, Slovakia, 63.3% etc.) [18]. In our study, the percentage of MDR *K*. *pneumoniae* was very high and varied between different hospital wards (S wards, 86.2% and M wards, 63.1%). These results are in the line with the annual Report of CAESAR network for 2013; among 30 EU-countries and some countries from ex-Yugoslavia, Serbia had the highest prevalence of MDR *K*. *pneumoniae* [3]. This is not the case in a tertiary care hospital from Nis, Serbia (1460 beds) where *P*. *aeruginosa* was more frequently isolated than *K*. *pneumoniae* [19]. But, their survey was carried out on different hospital wards (e. g. pediatric, otorhinolaryngology, neurology, orthopedic surgery, neurosurgery etc.). Moreover, Lee et al have found that in the Taichung Veterans Hospital in Taiwan, *P*. *aeruginosa* was more commonly isolated from 2003 up to 2011 than *K*. *pneumoniae* [2]. Our data are in accord with study of Datta et al. completed in a tertiary care hospital in New Delhi, where *K*. *pneumoniae* was a predominant organism in 2006 [20]. Reported ceftazidime and imipenem resistance rate of *K*. *pneumoniae* isolated on S wards is comparable with data reported by Datta et al. and Goel et al. [20, 21]. The observation that *K*. *pneumoniae* isolates from S wards had higher resistance rate to all tested antimicrobials in relation to M wards (Table 4) needs a special attention.

Avery high intra-country variation between national percentages of MDR *P*. *aeruginosa* (Estonia, 0%, Iceland, 0%, Slovenia, 22.3%, Bulgaria, 27.1%, Croatia, 30.0%, Greece, 37.7%, Romania, 59.6% etc.) was presented before. Unfortunately, we recorded higher percentage of MDR *P*. *aeruginosa* on our S (49.1%) and M (36.9%) wards than it was the case in the majority of EU countries but Romania [18]. Numerous worldwide studies reported the increasing prevalence of *P*.*aeruginosa* in different tertiary care hospitals between 1998–2011 [2, 21–23]. Fortunately, no significant increase of number of MDR *P*. *aeruginosa* was found on the relevant wards. In addition, Falagas et al. (Greece) showed no significant change in prevalence of MDR *P*. *aeruginosa* from 2002 to 2006 [24].

The prevalence and resistance rate of *A*. *baumannii* isolated in hospital settings increased significantly worldwide [25]. Our study showed that there was a significant increase in percentage of MDR *A*. *baumannii* on S (80% to 100%) and M wards (74% to 97%) from 2012 to 2015. Furthermore, in the countries with a very high percentage of MDR pathogens (Greece and Italy), the resistance rate of *A*. *baumannii* increased during the reporting period [18, 24]. A similar percentage (80%) of MDR *A*. *baumannii* was reported in a tertiary care hospital in New Delhi [21]. Our results show that there was a rapid development of *A*. *baumannii* resistance to ceftazidime, cefepime and imipenem as compared with *P*. *aeruginosa* (Table 4). The rate of resistance of *A*. *baumannii* to combined fluoroquinolone, aminoglycoside and carbapenem therapy in some European countries ranged from zero (Netherland, Denmark, Finland) to more than 80% (Greece, Italy, India) [21].

Despite its importance, there is very little available information about the consumption of antimicrobial agents in hospitals of Serbia. During four-year analysis, the highest total antibiotic consumption was observed in the first year (105.8 DBD on the S wards and 74.5 DBD on the M wards). In contrast to our data, Goel et al. during 10-year analysis (1999–2008), reported a significant increase in the antibiotic consumption in a tertiary care hospital in New Delhi, which doubled from 158.7 DBD to 318.5 DBD [21]. Furthermore, in another tertiary care hospital in New Delhi, Datta et al. demonstrated very high total antibiotic consumption, 226.5 DBD in 2009 [20]. Fortunately, a total antibiotic consumption in our wards in 2013–2015 did not increase and remained stable over time. In another large survey of 25 hospitals in the Mediterranean region, the antimicrobial consumption varied to a great extent, from 84 to 428 DBD, with median consumption of 112 DBD that was higher in comparison with our wards [26]. During the whole study interval, we found a significantly higher total antibiotic consumption on the S than on M wards, in spite of shorter mean hospital stay on the S wards. In contrast, Inan et al. showed that antibiotic prescription ratio in a teaching hospital in Istanbul, Turkey, was higher on the M (40.5%) than on the S wards (32.9%) [27]. Gentamicin, ceftriaxone, metronidazole, ciprofloxacin, and co-amoxiclav were five most frequently used antimicrobials on the S wards, while ceftriaxone, metronidazole, levofloxacin, co-amoxiclav and ciprofloxacin were those mostly used on the M wards (see Fig 3). As the most frequent diagnoses on the S wards were the urinary tract and gastrointestinal tract infections, the list of top five antimicrobials seemed to be appropriate [6–8]. Having in mind that the most frequent diagnoses on the M wards were respiratory tract and urinary tract infections, it is in accordance with the recommendations to treat these infections with ceftriaxone, fluoroquinolones and co-amoxiclav, but not with metronidazole [6, 7, 9]. In spite of higher percentage of recorded *C*. *difficile* infections, so high consumption of metronidazole on the M wards was not rational (see Table 2). High metronidazole consumption on the S wards may be in accordance with the international therapy guidelines for different intra-abdominal infections [10].

Our trend analysis has failed to show the correlation between a total antibiotic consumption and percentage of MDR *K*. *pneumoniae*, *P*. *aeruginosa* and *A*. *baumannii* strains on the S and M wards, and it might be due to small sample size (only 4 years of follow-up). Similarly, in study of Velickovic-Radovanovic et al. in spite of substantial increase in the use of ceftriaxone in a tertiary care hospital in Nis (Serbia) during 2005–2013, there was no significant trend towards increasing bacterial resistance [19].

In order to analyze the quality of antibiotic prescribing to hospitalized patients on the S and M wards, G-PPS method was applied for monitoring such prescribing [5]. Various prophylactic prescription practices were observed worldwide [4]. The percentage of antimicrobials administered for surgical prophylaxis (25%) on our S wards corresponded to preliminary data of Versporten et al. (25.3%), but it was higher (19.2%) than in the study of Cusini et al. [4, 17]. It is necessary to emphasize that over a half of antimicrobials was prescribed for prophylaxis (58.8%) more than one day, which was not in accordance with the local and international guidelines [6–10]. In addition, in several studies from Turkey, the authors disclosed high inappropriate prophylactic use of antibiotics (from 30%-50%) [27–29]. Empirical prescriptions were more often on our S and M wards (86.8% and 80%) than on the S and M wards of the University Hospital Zurich (47.4% and 67.2%) [17]. However, our data are comparable with report of Ozkurt et al. from the largest hospital in the Eastern Anatolian region of Turkey in 2001 [29]. Before the restriction policy, they reported that 62.2% and 86.3% of patients on the S and M wards, respectively, received empirical antimicrobial therapy, but 3 years upon the restriction policy, the percentage of patients with the empirical therapy decreased (63.2% and 68.7%) [29]. It is indisputable that we have to evaluate and improve our restriction policy.

There were remarkable differences in the patterns of inappropriate prescribing on the S and M wards (74.6% vs. 27.3%). Divergence from the local guidelines was the most important concern on the S wards (72.9%). In a prevalence survey of Cusini et al., divergence from the international guidelines on the S wards was only 0.9% [17]. But, as in our study, medical doctors on the S wards made errors more often than doctors on the M wards. Furthermore, inappropriate use of antibiotics (54.1%) is more typical for doctors on the S than on the M wards (32.5%) of tertiary hospital in Erzurm (Turkey) [29]. Major causes of antimicrobial misuse on our M wards were wrong dose and route of administration. In addition, we observed that biomarkers for establishing diagnosis of infection were more often used on the M wards than S wards. Indeed, these point-of-care tests seem to help doctors to identify who to treat or not treat with the antibiotics. For example, CRP rapid testing and PCT have been shown to significantly reduce antibiotic prescribing in lower respiratory infections [1]. The list of five most commonly prescribed antimicrobials on the M wards during G-PPS included ciprofloxacin, ceftriaxone, levofloxacin, metronidazole and azithromycin that was in accordance with the international guides recommending exactly this group of antibiotics for respiratory tract infections (CAP and HAP), and metronidazole for *C*. *difficile* infection [6, 7, 9]. As the most frequent reasons for antimicrobial prescription on the S wards were prophylaxis in the abdominal and urological surgery, and therapy of the surgical site infections and intervention-related infections (catheter-related blood stream infection, catheter-related urinary tract infection and ventilator associated pneumonia), the list of top five antimicrobials (gentamicin, ceftriaxone, amikacin, ciprofloxacin, and co-amoxiclav) seems to be appropriate [6–8].

## Conclusions

In conclusion, our study highlights alarming increase in resistance of *K*. *pneumoniae*, and *A*. *baumannii* on the S and M wards, in spite of all applied interventions for infection control as well as restriction and improvement of antibiotic use. The increasing prevalence of MDR pathogens on surgical wards needs a special attention. This problem is probably due to very high consumption of antimicrobials and incorrect antimicrobial prescribing.

This is the first study providing some information on consumption, prescribing and development of MDR hospital pathogens in Serbia. Limitations of this study are: i) short period of evaluation of antibiotic consumption and the incidence of MDR hospital pathogens; ii) information on one-day prescribing could not be correlated with trends in the proportion of antimicrobial resistance over four years. Accordingly, further evaluation to establish the correlation between prescribing and trends in the proportion of antimicrobial resistance is required.
